# Supplementary material for: Frequent exchange of the DNA polymerase during bacterial chromosome replication
Source: eLife. 2017 Mar 31;6:e21763. doi: 10.7554/eLife.21763 (PMC5403216; doi:10.7554/eLife.21763)
Supplement: Suplementary file 1. — (A) Analysis of FRAP data. (B) Analysis of sptPALM data. (C) Results of goodness-of-fit tests for sptPALM. DOI: http://dx.doi.org/10.7554/eLife.21763.016 [file elife-21763-fig1.docx]

**Supplementary file 1A. Analysis of FRAP data**

| **Strain** | **N** | **MaxR** | **A** | **Koff** | **TimeC** | **Asym** | **R sqrd** | **DoF** | **redChi** | **KS test** | **pKS** | **SE A** | **SE Koff** | **SE Asym** | **A 95% Conf** | **Koff 95% Conf** | **Asym 95% Conf** |
| --- | --- | --- | --- | --- | --- | --- | --- | --- | --- | --- | --- | --- | --- | --- | --- | --- | --- |
| **DnaB** | 96 | 0.749 | 0.312 | 0.135 | 7.388 | 0.483 | 0.835 | 19 | 0.015 | Nor | 0.876 | 0.059 | 0.051 | 0.016 | 0.131,0.343 | 0.018,0.213 | 0.455,0.514 |
| **DnaB** | 36 | 0.770 | 0.277 | 0.100 | 9.971 | 0.467 | 0.369 | 16 | 0.167 | Nor | 0.674 | 0.065 | 0.062 | 0.035 | 0.129,0.387 | 0.014,0.190 | 0.408,0.528 |
| **DnaB** | 25 | 0.823 | 0.225 | 3.540 | 0.282 | 0.484 | 0.204 | 22 | 0.092 | Nor | 0.440 | 0.018 | 0.000 | 0.018 | 0.189,0.262 | 3.54,3.54 | 0.450,0.521 |
| **DnaB +HU** | 35 | 0.793 | 0.189 | 0.122 | 8.165 | 0.388 | 0.775 | 10 | 0.011 | Nor | 0.985 | 0.056 | 0.058 | 0.024 | 0.082,0.358 | 0.022,0.232 | 0.355,0.417 |
| **α** | 44 | 0.924 | 0.795 | 0.272 | 3.677 | 0.907 | 0.665 | 24 | 0.111 | Nor | 0.458 | 0.072 | 0.065 | 0.039 | 0.698,0.890 | 0.155,0.399 | 0.834,0.984 |
| **α** | 48 | 0.783 | 0.564 | 0.240 | 4.172 | 0.699 | 0.942 | 8 | 0.014 | Nor | 0.920 | 0.112 | 0.072 | 0.059 | 0.415,1.07 | 0.134,0.455 | 0.622,0.753 |
| **α +HU** | 55 | 0.787 | 0.555 | 0.042 | 23.978 | 0.747 | 0.828 | 18 | 0.064 | Nor | 0.487 | 0.179 | 0.018 | 0.197 | 0.384,1.054 | 0.012,0.079 | 0.576,1.297 |
| **ß** | 54 | 0.762 | 0.454 | 0.027 | 37.341 | 0.670 | 0.749 | 31 | 0.070 | Nor | 0.117 | 0.044 | 0.008 | 0.060 | 0.387,0.536 | 0.007,0.038 | 0.629,0.866 |
| **ß** | 38 | 0.755 | 0.637 | 0.029 | 34.942 | 0.830 | 0.841 | 16 | 0.020 | Nor | 0.579 | 0.139 | 0.007 | 0.195 | 0.582,1.095 | 0.003,0.036 | 0.776,1.459 |
| **ß +HU** | 71 | 0.744 | 0.516 | 0.014 | 72.568 | 0.818 | 0.824 | 16 | 0.073 | Nor | 0.616 | 0.207 | 0.007 | 0.258 | 0.374,0.979 | 0.002,0.026 | 0.679,1.412 |
| **ε** | 17 | 0.734 | 0.435 | 0.068 | 14.666 | 0.808 | 0.682 | 14 | 0.200 | Nor | 0.997 | 0.159 | 0.039 | 0.183 | 0.267,0.827 | 0.011,0.129 | 0.666,1.288 |
| **ε** | 42 | 0.820 | 0.461 | 0.206 | 4.852 | 0.699 | 0.608 | 22 | 0.124 | Nor | 0.979 | 0.069 | 0.085 | 0.027 | 0.249,0.534 | 0.105,0.391 | 0.642,0.747 |
| **ε** | 71 | 0.729 | 0.523 | 0.167 | 5.999 | 0.771 | 0.804 | 22 | 0.032 | Nor | 0.335 | 0.058 | 0.036 | 0.027 | 0.365,0.598 | 0.108,0.273 | 0.725,0.828 |
| **ε** | 65 | 0.768 | 0.437 | 0.188 | 5.323 | 0.668 | 0.805 | 18 | 0.039 | Nor | 0.511 | 0.048 | 0.044 | 0.022 | 0.323,0.501 | 0.104,0.279 | 0.626,0.709 |
| **ε +HU** | 28 | 0.743 | 0.498 | 0.028 | 35.101 | 0.784 | 0.873 | 10 | 0.043 | Nor | 0.726 | 0.224 | 0.018 | 0.270 | 0.207,0.946 | 0.006,0.054 | 0.590,1.329 |
| **ε +HU** | 47 | 0.762 | 0.617 | 0.027 | 37.488 | 0.837 | 0.924 | 10 | 0.032 | Nor | 0.677 | 0.242 | 0.013 | 0.280 | 0.330,1.171 | 0.007,0.050 | 0.611,1.472 |
| **τ** | 43 | 0.721 | 0.487 | 0.235 | 4.247 | 0.662 | 0.617 | 22 | 0.068 | Nor | 0.997 | 0.075 | 0.059 | 0.029 | 0.410,0.576 | 0.158,0.390 | 0.607,0.719 |
| **τ** | 27 | 0.807 | 0.580 | 0.330 | 3.029 | 0.684 | 0.861 | 10 | 0.018 | Nor | 0.991 | 0.298 | 0.161 | 0.022 | 0.0580,1.102 | 0.033,0.627 | 0.640,0.724 |
| **τ** | 47 | 0.793 | 0.693 | 0.115 | 8.733 | 0.872 | 0.862 | 10 | 0.017 | Nor | 0.523 | 0.177 | 0.037 | 0.051 | 0.438,1.317 | 0.062,0.217 | 0.798,0.948 |
| **τ +HU** | 49 | 0.740 | 0.537 | 0.025 | 39.458 | 0.799 | 0.891 | 10 | 0.047 | Nor | 0.942 | 0.245 | 0.016 | 0.291 | 0.217,1.021 | 0.006,0.048 | 0.584,1.372 |
| **τ +HU** | 41 | 0.738 | 0.350 | 0.261 | 3.829 | 0.455 | 0.675 | 16 | 0.029 | Nor | 0.509 | 0.108 | 0.114 | 0.017 | 0.035,0.665 | 0.099,0.496 | 0.421,0.486 |
| **τ +HU** | 71 | 0.646 | 0.416 | 0.058 | 17.152 | 0.602 | 0.870 | 10 | 0.012 | Nor | 0.676 | 0.123 | 0.014 | 0.023 | 0.312,0.791 | 0.036,0.088 | 0.567,0.650 |
| **δ** | 48 | 0.847 | 0.757 | 0.292 | 3.428 | 0.932 | 0.816 | 21 | 0.056 | Nor | 0.718 | 0.093 | 0.120 | 0.028 | 0.314,0.823 | 0.130,0.554 | 0.887,0.993 |
| **χ** | 42 | 0.804 | 0.583 | 0.167 | 6.000 | 0.884 | 0.765 | 26 | 0.063 | Nor | 0.435 | 0.060 | 0.051 | 0.048 | 0.445,0.687 | 0.071,0.282 | 0.815,0.985 |

Each row represents an independent set of experiments

The model used for fitting the recovery curves was: $y=c-a\mathrm{e}^{-bt}$

where ‘a’ is the amplitude of recovery (**A**), ‘b’ is the rate of recovery (**Koff**) and ‘c’ is the asymptote for curve (**Asym**).

**MaxR**= Total cell intensity after bleaching step, defining the maximum possible recovery

Time Constant (**TimeC**) = 1/Koff

Degrees of Freedom (**DoF**) were defined as the number of time-points minus the number of parameters in the model

Kolmogorov-Smirnov (**KS**) test is used to determine if the distribution of the residuals is normal (Nor) as a measure of the goodness of fit

**pKS** is the p-value of the Kolmogorov-Smirnov test of the residuals

Standard Errors (**SE**) were calculated using Bootstrap resampling of the data with 10,000 iterations

95% Confidence values (**95% Conf**) were calculated from Bootstrap resampling

**Supplementary file 1B. Analysis of sptPALM data**

| **Strain** | **Exposure Time, Interval Time** | **Number of Molecules** | **Bound Time (s)** | **Std. Error (s)** | **95% Confidence Interval** |
| --- | --- | --- | --- | --- | --- |
| **ε** | 500ms,1 second | 40 | 4.0781 | 0.8499 | 2.6011 , 6.0413 |
| **ε** | 500ms,1 second | 143 | 7.4411 | 1.0731 | 5.3831,9.6147 |
| **ε** | 500ms,1 second | 57 | 5.3643 | 0.9316 | 3.7838,7.5324 |
| **ε** | 2seconds, 2seconds | 163 | 10.4058 | 1.9365 | 8.1133,16.5602 |
| **ε** | 2seconds, 2seconds | 415 | 12.3396 | 1.3616 | 10.2301,16.0835 |
| **ε** | 500ms,5 seconds | 106 | 12.2612 | 2.4566 | 8.4361 , 18.3918 |
| **ε** | 2 seconds, 10 seconds | 771 | 15.0272 | 1.2051 | 10.1861,31.5054 |
| **ε** | 2 seconds, 10 seconds | 307 | 16.5094 | 4.2964 | 12.6881,17.1335 |
| **ε** | 500ms,1 second | 145 | 10.8298 | 2.147 | 8.1978,16.9549 |
| **ε HU** | 500ms, 1 second | 94 | 22.2076 | 5.0136 | 13.2196,33.3087 |
| **ε HU** | 500ms, 5 seconds | 194 | 26.996 | 4.6829 | 19.5857 ,39.6222 |
| **ε RIF** | 500ms,1 second | 69 | 6.1274 | 1.4109 | 4.1338,10.2727 |
| **ε RIF** | 500ms,1 second | 60 | 7.081 | 2.8167 | 3.8225,19.7814 |
|  |  |  |  |  |  |
| **δ** | 500ms, 1 second | 78 | 11.5957 | 2.9982 | 6.8584, 17.3936 |
| **δ** | 500ms, 1 second | 92 | 8.697 | 1.5627 | 6.0750,12.4884 |
| **δ** | 500ms, 5 seconds | 139 | 13.5089 | 1.9453 | 10.3240 , 18.0131 |
| **δ** | 500ms, 5 seconds | 441 | 12.4475 | 1.2638 | 10.6088,16.0456 |
|  |  |  |  |  |  |
| **γ/τ** | 500ms, 1 second | 167 | 10.2711 | 1.54 | 7.5117, 13.5108 |
| **γ/τ** | 500ms, 1 second | 166 | 7.731 | 0.9497 | 6.7347,10.1444 |
| **γ/τ** | 500ms, 1 second | 64 | 10.0856 | 3.0839 | 5.5159 ,15.1284 |
| **γ/τ** | 500ms, 5 second | 276 | 11.0481 | 1.289 | 8.9082 ,14.0603 |
| **γ/τ** | 500ms, 5 second | 109 | 11.9405 | 2.0243 | 8.4691 16.7055 |
| **γ/τ HU** | 500ms, 1 second | 190 | 27.5391 | 4.1893 | 21.2238 , 40.0616 |
| **γ/τ HU** | 500ms, 5 second | 168 | 24.9486 | 3.7809 | 18.5154,33.7872 |
|  |  |  |  |  |  |
| **β** | 500ms, 1 second | 336 | 57.1851 | 15.5542 | 41.8224 ,82.1468 |
| **β** | 500ms, 1 second | 118 | 31.8378 | 4.6764 | 24.8192,44.4616 |
| **β** | 500ms, 1 second | 181 | 29.8311 | 7.1506 | 22.6254,59.9923 |
| **β** | 500ms, 5 seconds | 464 | 59.7503 | 6.0364 | 53.4034, 68.3717 |
| **β** | 500ms, 5 seconds | 273 | 41.2986 | 2.9074 | 35.7621, 49.6564 |
| **β** | 500ms, 5 seconds | 428 | 47.226 | 2.5643 | 42.8505,52.3607 |
| **β** | 500ms, 10 seconds | 178 | 38.8389 | 5.1799 | 30.1962,50.7123 |
| **β** | 500ms, 10 seconds | 263 | 43.0297 | 5.0548 | 34.324,54.2780 |
|  |  |  |  |  |  |
| **dnaB** | 500ms, 1 second | 81 | 58.6854 | 16.3232 | 31.8179, 88.0281 |
| **dnaB** | 500ms, 1 second | 39 | 43.9003 | 15.2807 | 21.9501, 65.8504 |
| **dnaB** | 500ms, 5 seconds | 60 | 37.7639 | 8.1653 | 24.1637, 56.6458 |
| **dnaB** | 2seconds,2seconds | 36 | 1681.2 | 2178.1 | 63.3,5395.3 |
| **dnaB** | 2seconds,2seconds | 57 | 1202.8 | 2103.6 | 99.8,5395.3 |
| **dnaB** | 2seconds,2seconds | 160 | 5285 | 1.93E+03 | 5035.4,5.3955 |
| **dnaB** | 2seconds,10 seconds | 124 | 407.5475 | 149.3787 | 217.7521,846.2488 |
| **dnaB** | 2seconds,10 seconds | 86 | 1864.6 | 1845.3 | 76.2,5398.1 |
| **dnaB** | 2seconds,10 seconds | 115 | 746.5645 | 365.5298 | 379.3,1720.8 |
| **dnaB HU** | 500ms, 1 second | 75 | 32.8548 | 6.7004 | 21.1815 , 48.1302 |
| **dnaB HU** | 500ms, 5 seconds | 95 | 69.0107 | 16.8591 | 41.1344,103.5161 |

**Supplementary file 1C. Results of goodness-of-fit tests for sptPALM**

| **Strain** | **Exposure Time, Interval Time** | **Number of Molecules** | **Chi square (p<0.01)** | **Likelihood ratio Test (p<0.01)** | **BIC Test (Single vs Double)** | **Talpha (s)** | **Tbeta (s)** |
| --- | --- | --- | --- | --- | --- | --- | --- |
| **ε** | 500ms,1 second | 40 | 0.7482 | 7.41E-02 | 184.6894 188.8769 | 7.7879 | 1.776 |
| **ε** | 500ms,1 second | 143 | 0.497 | 1.29E-06 | 777.7910 764.2785 | 14.5014 | 1.5326 |
| **ε** | 500ms,1 second | 57 | 0.3432 | 0.1911 | 285.9711 292.3479 | 6.9698 | 1.4735 |
| **ε** | 2seconds, 2seconds | 163 | 0.1035 | 0 | 1.0531 0.8344*10^3 | 16.8438 | 0.1 |
| **ε** | 2seconds, 2seconds | 415 | 0.0149 | 0 | 2.7687 2.6880*10^3 | 32.4254 | 4.3654 |
| **ε** | 500ms,5 seconds | 106 | 0.5092 | 0.00E+00 | 727.6712 474.5390 | 22.8176 | 0.1 |
| **ε** | 2 seconds, 10 seconds | 771 | 3.81E-27 | 0 | 5.6370 2.3824*10^3 | 34.5085 | 0.1 |
| **ε** | 2 seconds, 10 seconds | 307 | 0.0245 | 0 | 2.2915 1.0299*10^3 | 37.4506 | 0.1 |
| **ε** | 500ms,1 second | 145 | 0.4957 | 1.82E-10 | 797.3873 766.6519 | 11.3787 | 0.1 |
| **ε HU** | 500ms, 1 second | 94 | 0.3673 | 1.64E-08 | 637.4230 614.6339 | 43.8949 | 0.1 |
| **ε HU** | 500ms, 5 seconds | 194 | 0.0486 | 0.00E+00 | 1.5828 1.2822*10^3 | 43.6251 | 0.1 |
| **ε RIF** | 500ms, 1 second | 69 | 0.9248 | 0.0484 | 336.9174 341.4590 | 84.3264 | 4.1604 |
| **ε RIF** | 500ms, 1 second | 60 | 0.0265 | 0.0054 | 264.7774 265.1736 | 10.738 | 1.9023 |
|  |  |  |  |  |  |  |  |
| **δ** | 500ms, 1 second | 78 | 0.007 | 6.96E-07 | 474.4378 458.5241 | 2.94E+03 | 4.2 |
| **δ** | 500ms, 1 second | 92 | 0.4768 | 4.56E-14 | 523.3928 475.5273 | 13.6277 | 0.1 |
| **δ** | 500ms, 5 seconds | 139 | 0.3431 | 0.00E+00 | 975.6567 686.1073 | 23.426 | 0.1 |
| **δ** | 500ms, 5 seconds | 441 | 2.17E-08 | 0.00E+00 | 3.0126 2.2388*10^3 | 19.8989 | 0.1 |
|  |  |  |  |  |  |  |  |
| **γ/τ** | 500ms, 1 second | 167 | 0.3493 | 1.99E-08 | 981.3559 960.0837 | 39.6638 | 2.8935 |
| **γ/τ** | 500ms, 1 second | 166 | 0.1804 | 0.00E+00 | 910.7361 831.9867 | 11.4531 | 0.1 |
| **γ/τ** | 500ms, 1 second | 64 | 0.0469 | 4.03E-10 | 378.9490 348.1694 | 5399.9 | 4.1 |
| **γ/τ** | 500ms, 5 second | 276 | 0.0968 | 0.00E+00 | 1.8303 1.2212*10^3 | 19.554 | 0.1 |
| **γ/τ** | 500ms, 5 second | 109 | 0.1869 | 0.00E+00 | 742.9151 495.1825 | 21.6465 | 0.1 |
| **γ/τ HU** | 500ms, 1 second | 190 | 0.0104 | 2.45E-11 | 1.3167 1.2826 *10^3 | 2.73E+03 | 3.9 |
| **γ/τ HU** | 500ms, 5 second | 168 | 9.29E-04 | 0.00E+00 | 1.3508 1.0929*10^3 | 39.8463 | 0.1 |
|  |  |  |  |  |  |  |  |
| **β** | 500ms, 1 second | 336 | 0.0028 | 0 | 2.4845 2.4201 *10^3 | 5400 | 6.4 |
| **β** | 500ms, 1 second | 118 | 0.7531 | 2.75E-11 | 874.4232 839.6167 | 65.93 | 0.1 |
| **β** | 500ms, 1 second | 181 | 0.2842 | 5.44E-15 | 1.2309 1.1801*10^3 | 105.3916 | 0.1 |
| **β** | 500ms, 5 seconds | 464 | 1.45E-08 | 0.00E+00 | 4.2937 3.6104 *10^3 | 116.3723 | 0.1 |
| **β** | 500ms, 5 seconds | 273 | 0.0031 | 0 | 2.4390 2.0324*10^3 | 65.0855 | 0.1 |
| **β** | 500ms, 5 seconds | 428 | 1.89E-04 | 0 | 3.7845 3.2143*10^3 | 86.8245 | 0.1 |
| **β** | 500ms, 10 seconds | 178 | 0.0793 | 0 | 1.6199 1.1354*10^3 | 63.1876 | 0.1 |
| **β** | 500ms, 10 seconds | 263 | 0.0013 | 0 | 2.3954 1.7464*10^3 | 70.2949 | 0.1 |
|  |  |  |  |  |  |  |  |
| **dnaB** | 500ms, 1 second | 81 | 0.0233 | 5.54E-07 | 605.9887 589.7109 | 5400 | 3.5 |
| **dnaB** | 500ms, 1 second | 39 | 0.5106 | 6.32E-06 | 288.5588 275.4979 | 1049 | 0.1 |
| **dnaB** | 500ms, 5 seconds | 60 | 0.0235 | 0.00E+00 | 524.7951 427.8399 | 67.4397 | 0.1 |
| **dnaB** | 2seconds,2seconds | 36 | N/A | 1.92E-05 | 358.2261 347.1240 | 5400 | 7 |
| **dnaB** | 2seconds,2seconds | 57 | 0.0879 | 1.53E-09 | 562.4826 534.0724 | 1353.3 | 0.1 |
| **dnaB** | 2seconds,2seconds | 160 | 0.0089 | 0 | 1.5839 1.5203*10^3 | 5400 | 4.1 |
| **dnaB** | 2seconds,10 seconds | 124 | 2.28E-04 | 0 | 1.4453 1.1710*10^3 | 5.3992 | 0.1 |
| **dnaB** | 2seconds,10 seconds | 86 | 0.1985 | 0 | 1.0963 0.8884 *10^3 | 5400 | 0.1 |
| **dnaB** | 2seconds,10 seconds | 115 | 0.0258 | 0 | 1.3971 1.1542*10^3 | 5381.5 | 0.1 |
| **dnaB HU** | 500ms, 1 second | 75 | 0.0796 | 1.17E-11 | 534.6678 497.2866 | 161.2 | 0.1 |
| **dnaB HU** | 500ms, 5 seconds | 95 | 0.0013 | 0.00E+00 | 902.2316 764.1085 | 147.6445 | 0.1 |
|  |  |  |  |  |  |  |  |
| **ε** | 500ms,1 second | 142 | 0.5658 | 2.89E-15 | 750.1909 697.7604 | 9.3415 | 0.1 |
| **ε** | 2seconds, 2seconds | 413 | 0.0136 | 0.00E+00 | 2.7043 2.3847 *^3 | 16.3533 | 0.1 |
| **δ** | 500ms, 1 second | 76 | 0.1063 | 6.18E-06 | 421.5906 409.8209 | 10.305 | 0.1 |
| **γ/τ** | 500ms, 1 second | 161 | 0.4302 | 1.02E-12 | 877.2507 836.6067 | 10.1318 | 0.1 |
| **γ/τ HU** | 500ms, 1 second | 186 | 0.0979 | 6.24E-13 | 1.2295 1.1882 *10^3 | 33.771 | 0.1 |

***Data sets highlighted in yellow indicate ones that initially suggested a two -exponential model. Data highlighted in green represents the results after outliers were removed from these data sets. The difference in the number of molecules indicates the number of outliers removed.**
